# Blob-B-Gone: a lightweight framework for removing blob artifacts from 2D/3D MINFLUX single-particle tracking data

**DOI:** 10.3389/fbinf.2023.1268899

**Published:** 2023-11-22

**Authors:** Bela T. L. Vogler, Francesco Reina, Christian Eggeling

**Affiliations:** ^1^ Leibniz Institute of Photonic Technology e.V., Member of the Leibniz Centre for Photonics in Infection Research (LPI), Jena, Germany; ^2^ Institute of Applied Optics and Biophysics, Faculty of Physics and Astronomy, Friedrich Schiller University Jena, Jena, Germany; ^3^ Jena Center for Soft Matter, Friedrich Schiller University Jena, Jena, Germany; ^4^ Abbe Center of Photonics, Friedrich Schiller University Jena, Jena, Germany

**Keywords:** artifact removal, MINFLUX, single-particle tracking, clustering, annotation, point clouds, geometry

## Abstract

In this study, we introduce Blob-B-Gone, a lightweight framework to computationally differentiate and eventually remove dense isotropic localization accumulations (blobs) caused by artifactually immobilized particles in MINFLUX single-particle tracking (SPT) measurements. This approach uses purely geometrical features extracted from MINFLUX-detected single-particle trajectories, which are treated as point clouds of localizations. Employing *k-means++* clustering, we perform single-shot separation of the feature space to rapidly extract blobs from the dataset without the need for training. We automatically annotate the resulting sub-sets and, finally, evaluate our results by means of principal component analysis (PCA), highlighting a clear separation in the feature space. We demonstrate our approach using two- and three-dimensional simulations of freely diffusing particles and blob artifacts based on parameters extracted from hand-labeled MINFLUX tracking data of fixed 23-nm bead samples and two-dimensional diffusing quantum dots on model lipid membranes. Applying Blob-B-Gone, we achieve a clear distinction between blob-like and other trajectories, represented in F1 scores of 0.998 (2D) and 1.0 (3D) as well as 0.995 (balanced) and 0.994 (imbalanced). This framework can be straightforwardly applied to similar situations, where discerning between blob and elongated time traces is desirable. Given a number of localizations sufficient to express geometric features, the method can operate on any generic point clouds presented to it, regardless of its origin.

## 1 Introduction

When performing single-particle tracking (SPT) experiments using optical microscopy, the vast majority of methodologies require some form of labeling to enable single-molecule detection, be it fluorescent tags or some form of highly scattering tag for scattering-based detection (Manzo and Garcia-Parajo, 2015). In a concrete experimental setting, the labeling procedure inevitably leads to several side effects, such as unbound tags or cross-linked reporters being present as immobile objects in the field of view. These artifacts may, in turn, lead to large accumulations of localizations in small regions, hereinafter defined as blobs, which may disturb the final detection. This is especially unwanted in cases where the diffusing species of interest undergoes transient confinements or trapping, rendering the detection of these behaviors especially complex.

Recently developed MINFLUX microscopy (Balzarotti et al., 2017; Gwosch et al., 2020; Schmidt et al., 2021) has shown great potential for high-throughput single-particle tracking (SPT) in two- and three-dimensions of single fluorescently tagged molecules. Due to the nature of its implementation, especially concerning its commercial version, a MINFLUX microscope detects all fluorescent reporters in a pre-defined region of interest (ROI), producing an array of coordinates and corresponding time stamps. Thus, immobile markers have a higher chance of being tracked in MINFLUX multiple times compared to the freely moving particles, which diffuse in and out of the ROI. In the case of SPT, it is preferable to use reporters that may be tracked for extended periods of time, such as metallic core quantum dots or, especially, photostable fluorescent dyes. Together with the core concept of MINFLUX of producing localization with comparatively few photons, the presence of immobile particle artefacts may be accentuated.

Due to the single-digit nanometer resolution of MINFLUX microscopy, the size of markers is a non-negligible factor in MINFLUX data. In general, we observe that immobile particles appear as either circular or spherical isotopically distributed point clouds with their radius proportional to the size of the marker. In the following work, we will refer to these spherical artifacts as blobs. These blobs need to be removed from the dataset before any analysis of particle motion can take place, as they can drastically influence the analysis of specific cases of particle diffusion, such as those characterized by transient confinements or “hopping” behavior (Kusumi et al., 2005; Honigmann et al., 2013). Classically, sorting is done by hand or by means of conventional statistical diffusion analysis and outlier reduction. This, however, becomes significantly more costly in computation time and power when applied to large quantities of long trajectories produced by high-throughput techniques such as MINFLUX. Recent AI-enabled tools for classification and clustering of particle trajectories are able to deliver promising results but require heavy computational power, large databases for training, and are specific in their application (Muñoz-Gil et al., 2020).

We propose a lightweight solution to this issue that is based on point cloud geometry and is able to rapidly identify and sort out all blob-like particle trajectories in two- and three-dimensional SPT data. Though this application was designed with MINFLUX in mind, it can, in essence, be transferred and applied to any point cloud-based technique, given that one sub-population of data exhibits blob-like behavior.

## 2 Methodology

The following section will introduce and describe the methods used to remove blobs from point cloud datasets. First, we investigate deliberately created MINFLUX SPT blobs to understand their geometry. Following that, we describe how we simulated and diversified diffusion and blob datasets in two and three dimensions to attain more rigid test samples. We use untrained single-shot *k-means clustering* (David Arthur, 2007) based on five designed classical geometrical features, which are calculated for each set independently. Lastly, we give a brief explanation of why we chose them and how they are computed.

### 2.1 MINFLUX blob artifacts

A single immobile bead sample was used to collect examples of blob-like structures using MINFLUX microscopy. This is a GATTA-Beads “R” sample (*GATTAquant* GmbH), purchased as a pre-mounted standard microscope slide from the producer. The beads have a nominal diameter of 23 nm (verified by STED microscopy) and are filled with ATTO 647N dye.

The beads were imaged using a commercial MINFLUX setup (*abberior* GmbH), which is based on an iterative localization approach (Schmidt et al., 2021). The setup used for the measurements reported herein is comprised of a confocal and MINFLUX illumination/detection unit attached to an Olympus IX83 microscope body. The illumination and detection objective is a ×100 oil immersion objective lens (UPL SAPO100XO/1.4, Olympus). For our MINFLUX measurements, we used a 642 nm excitation line, and the microscope detects the fluorescence with two avalanche photodiodes with detection ranges of 650–685 nm and 685–760 nm to estimate the localization of the fluorescence emitters in two dimensions (for the experiments contained herein). Photon detection takes place in a confocal microscopy fashion through a pinhole size corresponding to 1.0 AU. The localizations are derived from the sum of the photons from the two samples. Hardware control is provided using a version of *Imspector* software (*abberior* GmbH) that supports MINFLUX detection.

The *abberior* MINFLUX localizes particles with an iterative scanning approach with a pre-defined sequence of iterations (Gwosch et al., 2020; Schmidt et al., 2021). In brief, the microscope initially detects a fluorescence signal in an area by scanning a donut-shaped beam in a certain number of positions in an orbit with a radius L in various positions in a small field of view. Subsequently, the microscope “closes in” on the fluorescence signal by reducing the radius L and refining the position estimation. Each successive iteration also requires a different number of photons to produce localizations and can have a different dwell time and excitation laser power, as reported in the parameter table. When the microscope reaches the last iteration of the sequence, the system is locked on the latest detected particle and continues localizing it with the same parameters as long as photons are detected or the particle is detected within the scanning orbit [through the center frequency ratio (CFR) metric (Schmidt et al., 2021)].

In the case of a diffusing fluorescent molecule, the position update serves as a direct tracking method, delivering particle trajectories out of the microscope without additional particle tracking steps, as it is more usual for SPT through other microscopy techniques.

Essential parameters for the scanning sequences used, which are optimized for SPT, are listed in Tables 1–3 for immobile two- and three-dimensional as well as two-dimensional mobile tracking, respectively. We need to highlight that another variable that changes between pattern iterations is the excitation laser power. We express this by listing a laser power multiplier amongst the parameters, which refers to a reference excitation power of 1.78 μW at the sample plane.

**TABLE 1:** MINFLUX scanning parameters for 2D tracking of immobile particles.

2D tracking	First iteration	Second iteration	Third iteration	Fourth iteration
L (nm)	284	302	150	100
Pattern shape	Hexagon	Hexagon	Hexagon	Hexagon
Collected photons (counts)	40	20	20	20
Laser power multiplier (times)	1	1	2	3
Pattern dwell time (µs)	100	100	100	100
Pattern repeat (times)	1	1	1	1
Center frequency ratio (CFR)	−1.0	−1.0	0.8	−1
Background threshold (kHz)	15	30	30	50

**TABLE 2:** MINFLUX scanning parameters for 3D tracking of immobile particles.

3D tracking	First iteration	Second iteration	Third iteration	Fourth iteration	Fifth iteration
L (nm)	285	1,440	285	150	100
Pattern shape	Hexagonal (lateral localization)	Z line shape (axial localization)	Octahedron	Octahedron	Octahedron
Collected photons (counts)	40	300	40	30	30
Laser power multiplier	1	1	1	2	3
Pattern dwell time (µs)	500	2000	200	200	200
Pattern repeat (times)	1	1	1	1	1
Center frequency ratio (CFR)	−1	−1	−1	0.9	−1
Background threshold (kHz)	30	30	35	40	60

**TABLE 3:** MINFLUX scanning parameters for 2D tracking of mobile particles.

2D tracking	First iteration	Second iteration	Third iteration	Fourth iteration
L (nm)	282	300	150	100
Pattern shape	Hexagon	Hexagon	Hexagon	Hexagon
Collected photons (counts)	40	20	20	20
Laser power multiplier (times)	6.8	6.8	13.44	19.86
Pattern dwell time (µs)	100	100	100	100
Pattern repeat (times)	1	1	1	1
Center frequency ratio (CFR)	−1.0	−1.0	0.8	−1
Background threshold (kHz)	15	30	30	50

#### 2.1.1 Reconstructing blobs

**FIGURE 1:**
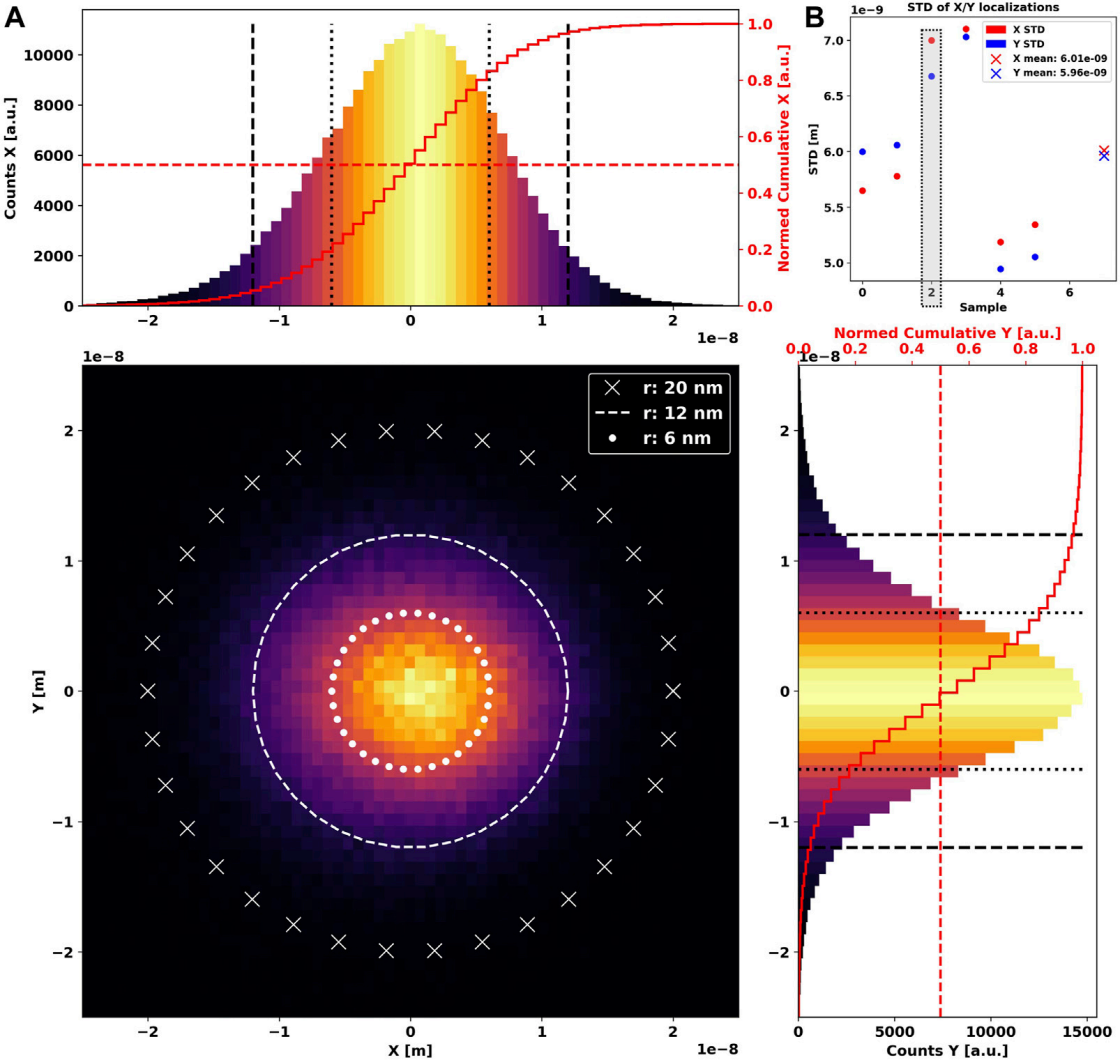
Two-dimensional histogram of 239,041 localizations **(A)** of an immobile *GATTAquant* 23-nm bead, acquired using MINFLUX single-particle tracking over 36 s. Three white circles are shown additionally for reference. The X-axis profile is shown as a 1D histogram in the upper panel, and the Y-axis profile is in the right-side panel. A common full red line in both graphs marks the cumulative histogram, i.e., the total count of localizations until the respective coordinate. The dotted red line highlights 50% of all localizations. The extracted standard deviations in *X* and *Y* directions for the entire dataset is shown in **(B)**. Crosses mark the mean value of X/Y standard deviation. A gray box highlights the dataset used in **(A)**.

Using MINFLUX tracking data of immobile fluorescent beads as a base model, we attempt to empirically recreate blobs *in silico* as the backbone for later simulations. Initially, we inspect the spread of localizations of an arbitrarily chosen MINFLUX blob using a two-dimensional histogram (Figure 1A central panel). We further show the respective distributions along X and Y, which reveals a clear center-symmetrical Gaussian shape (Figure 1A side panels).

To understand the expanse of the localization dispersion, we extract standard deviations along all axes for every dataset taken and show them grouped by sample in Figure 1B. It is immediately apparent that they are close but not identical, indicating a slightly elliptical shape, which we have to consider during simulation.

We further note that approximately 50% of localizations are found within a radius of 6 nm, while the overwhelming majority are located within the bounds of 12 nm from the center. Following these observations, we generate synthetic blobs as multivariate two- or three-dimensional normal distributions (Supplementary Figure S1).

While we can arbitrarily choose mean values to spawn the blobs across a sandbox, we need to give a covariance matrix which describes the distributions’ extent in space. As we observe the blobs to be concentric and isotropic, we will simplify the covariance matrix across all simulations to be diagonalized, meaning that all axes are independent of one another. The entries of said diagonal matrices are based on the standard deviations extracted from observation (Supplementary Figure S2).

#### 2.1.2 MINFLUX SPT data

After verifying the effectiveness of the proposed algorithm when applied to ground truth simulated data, we additionally acquired a set of MINFLUX SPT data to serve as our reference set when applying the artifact removal to real-world conditions.

To this end, we prepared giant unilamellar vesicles (GUVs) through electroformation using a solution of POPC:Chol 1:1 with DSPE-PEG20k-Biotin $\left(0.01Mol\%\right)$ and DOPE Atto 488 $\left(0.01Mol\%\right)$ , similar to the process of Méléard et al. (2009). We then plasma-cleaned a 25−mm coverslip to rapture the GUVs and create GUV patches. We used 1ml of phosphate-buffered saline (PBS, 137mM NaCl, 10mM phosphate, and 2.7mM KCl) to keep the supported lipid bilayer (SLB) hydrated. Finally, we labeled the biotinylated lipid in the SLB with 2μl of Qdot 655 streptavidin conjugate ( 10nM concentration, Invitrogen by Thermo Fisher Scientific) to perform SPT.

Multiple two-dimensional datasets have been taken on the same sample at different spots to increase variability and statistical rigidity. In the end, all sub-sets were pooled together, and any track or blob with less than 500 localizations was discarded to eradicate hardly analyzable trace fragments. Thus, both the artifact and free set range from ∼500 to ∼5,000 localizations.

Consecutively, all trajectories have been hand-labeled to create a reference dataset to benchmark the method. The set includes 107 blobs and 528 mobile tracks, which indicates on average the presence of approximately 20% artifacts per dataset.

### 2.2 Simulation

#### 2.2.1 Simulated ground truth datasets

As a ground truth dataset to validate our blob identification algorithm, we generated simulated datasets of blobs and diffusing particles. To represent particle diffusion, we simulated 250 traces of a variable number of localizations (between 400 and 600), with a time interval between localizations of Δt=500μs between steps. To introduce a degree of variability in the dataset, the diffusion coefficient of each simulated trajectory was randomly drawn from a uniform distribution of diffusion coefficients in the range of $\left[0.1\mu \right.m^{2}/s,$ $1\mu m^{2}\left./s\right]$ .

Each particle started at a position randomly chosen within a sandbox ( 1μm x 1μm ). We updated each localization ( $x_{i},y_{i}$ ) stepwise and simultaneously for all particles following $x_{i+1}=x_{i}+rcos\left(\varphi \right)$ and $y_{i+1}=y_{i}+rsin\left(\varphi \right)$ with $r=u\cdot \sqrt{2D\cdot \Delta t}\left|u\in \right.\left[0,1\right]$ and $\varphi \in \left[0,2\pi \right]$ . In doing so, we ensured being as close as possible to thermodynamic movement while maintaining idealized simplicity.

Blobs are created by drawing 400 to 600 localizations from a set of 250 two-dimensional multivariate normal distributions, where the covariance matrices were diagonalized, and the respective entries are again drawn from 1D normal distributions based on the mean and standard deviation of variances extracted from the MINFLUX tracking data of immobilized fluorescent beads. We did so to ensure that our simulation represents the variability of experimental data. Artifacts are randomly spawned in an area smaller than the area initially explored by synthetic free particles to avoid edge effects.

Ground truth datasets for the three dimensions are similarly generated, with the added degree of freedom of the third dimension.

### 2.3 Geometrical features

From our observations and knowledge of the origins of blobs, we can infer clear features that can be systematically detected in all blobs, highlighting their similarities. The better we describe them, the more similar descriptors they will produce, leading to a closer and higher density distribution in the feature space, aiding us when clustering.

#### 2.3.1 Maximum distance

A straightforward metric to distinguish between blobs originating from immobile particles and freely diffusing particles is to calculate the maximum Euclidean distance between any two points within the dataset. It will be similar for all blobs in the sample; however, it varies and is potentially larger for free markers. We can define the maximum distance as

$$d_{\max}=\max\left(\left\{\left|\vec{a}-{\vec{x}}_{i}\right|\right\}\forall \vec{a}\in P,\forall {\vec{x}}_{i}\in P\backslash \left\{\vec{a}\right\}\right),$$ (1)

where P is the set of points in each trajectory.

#### 2.3.2 Convex hull volume and area

We can assume the area or volume explored by any particle to be described by the area or volume of either a 2D polygon or 3D polyhedron with boundaries defined by the convex closure of the track, i.e., the smallest set that contains it as a subset of the Euclidean space. While blobs will result in regular spherical shapes with comparable area and volume throughout the dataset, free particles produce elongated or otherwise irregular shapes with varying parameters. We compute the convex hull using the *Qhull* algorithm (Bradford Barber et al., 1996), as implemented in the *SciPy* (Virtanen et al., 2020) package of Python, which we also use to calculate both the polygon area and polyhedron volume.

#### 2.3.3 Ellipticity

For non-blobs, the convex closure polygon appears as a stretched ellipsis due to the free movement of the particle if at least one direction is generally preferred, which is usually the case. Compared to that, a particle would need to constantly move in a concentric spiral or otherwise convex isometric around its starting position to produce a sphere or circle, which is possible but of low probability. We compare the convex area to the ellipsis calculated using the maximum Euclidean distance (1) and mean Euclidean distance to the point cloud’s center of mass.

$$ELLI_{2D}=\frac{A_{convex}}{A_{ellipsis}},$$ (2)

$$ELLI_{3D}=\frac{V_{convex}}{V_{ellipsoid}},$$

with

$$A_{ellipsis}=\pi \cdot d_{\max}\cdot d_{mean}=\pi {\cdot d}_{\max}\cdot \bar{\vec{a}-\vec{\mu }}\left|\mu =\right.\bar{P}.$$ (3)

The same is applied for comparing the volume of the convex polyhedron to the volume of an ellipsoid using the following expression:

$$V_{ellispoid}=\frac{4\pi }{3}\cdot d_{\max}\cdot d_{mean}^{2}.$$ (4)

#### 2.3.4 Center sphericality

Due to the isotropic distribution of localizations around the center of mass expected in blobs, the convex polygon resembles a circle, and the polyhedron resembles a sphere. Due to the bell-curve-shaped density profile along each axis, we expect and observe a significant difference between the convex closure and the area or volume described by a circle or sphere with the radius equal to the mean distance of each point to the center of mass. We effectively compare the area or volume of the highest density to the territory explored in total. In parallel to the ellipticity (2), we define the center sphericality as follows:

$$SPHE_{2D}=\frac{A_{convex}}{A_{circle}},$$ (5)

$$SPHE_{3D}=\frac{V_{convex}}{V_{sphere}}.$$

#### 2.3.5 Convex density of points

Based on the convex polygon area and convex polyhedron volume, respectively, we calculate the density of localizations in the total area and volume explored, highlighting fast and freely moving particles with a low density and separating them from densely packed blob tracks.

$$\rho_{2D}=\frac{{\#}_{P}}{A_{convex}},$$ (6)

$$\rho_{3D}=\frac{{\#}_{P}}{V_{convex}}.$$

### 2.4 Evaluation metrices

#### 2.4.1 F1-score

The F1-score is a widely used metric in statistical analysis of binary classification to evaluate accuracy. It is the harmonic mean of precision and recall (Taha and Hanbury, 2015) and thus represents both simultaneously. It is calculated as follows:

$$F1=2\cdot \frac{\text{precision}\cdot \text{recall}}{\text{precision}+\text{recall}}=\frac{2\cdot TP}{2\cdot TP+FP+FN},$$ (7)

where TP is the true positive, FP is the false positive, and FN is the false negative of the separation. F1-score values are in the range of [0,1], where larger values mean higher accuracy.

#### 2.4.2 Silhouette score

Performing cluster evaluation on the model itself, the silhouette score S (Rousseeuw, 1987) compares the mean distance between any selected sample and all other points within the cluster $D_{i}$ and the mean distance of any selected sample of one class to the next nearest cluster $D_{i+1}$ to quantify the sharpness of separation. It is calculated as follows:

$$S=\frac{D_{i+1}-D_{i}}{\max\left(D_{i},D_{i+1}\right)}.$$ (8)

The silhouette score yields values in the range of [−1,1], where larger values indicate clean separation of clusters.

#### 2.4.3 Adjusted Rand index

The similarity of label assignment can be quantified using the Rand index (Hubert and Arabie, 1985), a symmetrical measure between the number of pairs that are both in the same ground truth and clustered set A as well as pairs that are in different ground truth and clustered sets B . To set a common baseline for reliable interpretation, the Rand index is adjusted (Steinley, 2004) so that it yields 0 for random labeling and 1 for consistent labeling. The adjusted Rand index ARI is calculated as follows:

$$\text{ARI}=\frac{\text{RI}-E\left[\text{RI}\right]}{\max\left(\text{RI}\right)-E\left[\text{RI}\right]}.$$ (9)

The Rand index RI is defined as follows:

$$\text{RI}=\frac{A+B}{C_{2}^{n_{samples}}},$$ (10)

for the ground truth class assignment C .

#### 2.4.4 V-measure

Given the ground truth, the V-measure is the harmonic mean of clustering *homogeneity* and *completeness* (Andrew Rosenberg J. V-measure, 2007), representing both in one combined metric. It is calculated as follows:

$$V=\frac{\left(1+\beta \right)\cdot \text{homogeneity}\cdot \text{completeness}}{\beta \cdot \text{homogeneity}+\text{completeness}},$$ (11)

where β is a weight parameter set to 1 in this work.

The values are derived in the range of [0,1], where 1 is produced when each cluster contains only one species (homogeneity), and all candidates are assigned their ground truth label.

#### 2.4.5 Feature correlation

We evaluate the correlation between two feature axes by means of Pearson’s correlation coefficient (PCC) (Weisstein, 2023) as the ratio between the axes’ covariance and the product of their standard deviation. This yields a measure for the linear relationship between said features on a scale of [−1,1], where the extrema indicate ideal anti-correlation/correlation, respectively. PCC is calculated as follows:

$${\text{PCC}}_{F_{1},F_{2}}=\frac{cov\left(F_{1},F_{2}\right)}{\sigma_{F_{1}}\sigma_{F_{2}}}.$$

### 2.5 Automatic annotation

#### 2.5.1 Blob score

When applying Blob-B-Gone to unknown datasets without prior training, we lack control over the assignment of blobs to clusters during the process. Though visual confirmation by plotting is a possibility, it is hardly scalable and prone to bias. Thus, we introduce the Blob score B , which is the mean ratio between *central sphericality* and *maximum distance*, computed for both clusters to determine the set that is more likely to contain artifactual blobs:

$$B_{cluster}=\bar{SPHE/d_{\max}}.$$ (12)

We expect small distances and large center sphericality for artifacts, resulting in larger B -scores, and *vice versa* for the free tracks. Hence, using this metric, we automatically annotate both divided sub-sets fully automatically to make further processing easy.

We deliberately do not use the Blob score as a means of clustering since it is highly specific and, thus, has a higher bias toward a limited type of data, which results in diminished reliability when presented with inputs diverting from that.

## 3 Results

### 3.1 Simulated data

For each trace in the simulated datasets, we calculate a five-dimensional descriptor in the feature space using the features described in the Methodology section. Due to the considerable difference in the value across all features, the descriptors span an inhomogeneous space. To correct for that, we normalize and standardize all axes individually to match a zero-mean unit-variance distribution, thus removing feature bias and improving the rigidity of clustering. In order to evaluate the performance of the algorithm, we generate ground truth datasets by combining simulated blobs and freely diffusing particle trajectories, both in two and three dimensions. The chosen geometrical features are calculated for each of these point clouds, and we subsequently initialize k-means clustering with two suspected underlying populations. We compute the separation using the *scikit-learn* (Pedregosa et al., 2011) (Python) implementation of the *greedy k-means++* (David Arthur, 2007) algorithm in a single-shot manner, without the need for prior training.

To quantify the performance of the algorithm, we calculate the silhouette score for goodness of clustering, the adjusted Rand index to evaluate the labeling, the V-measure to check cluster formation, and the F1-score in addition to the confusion matrix.

**FIGURE 2:**
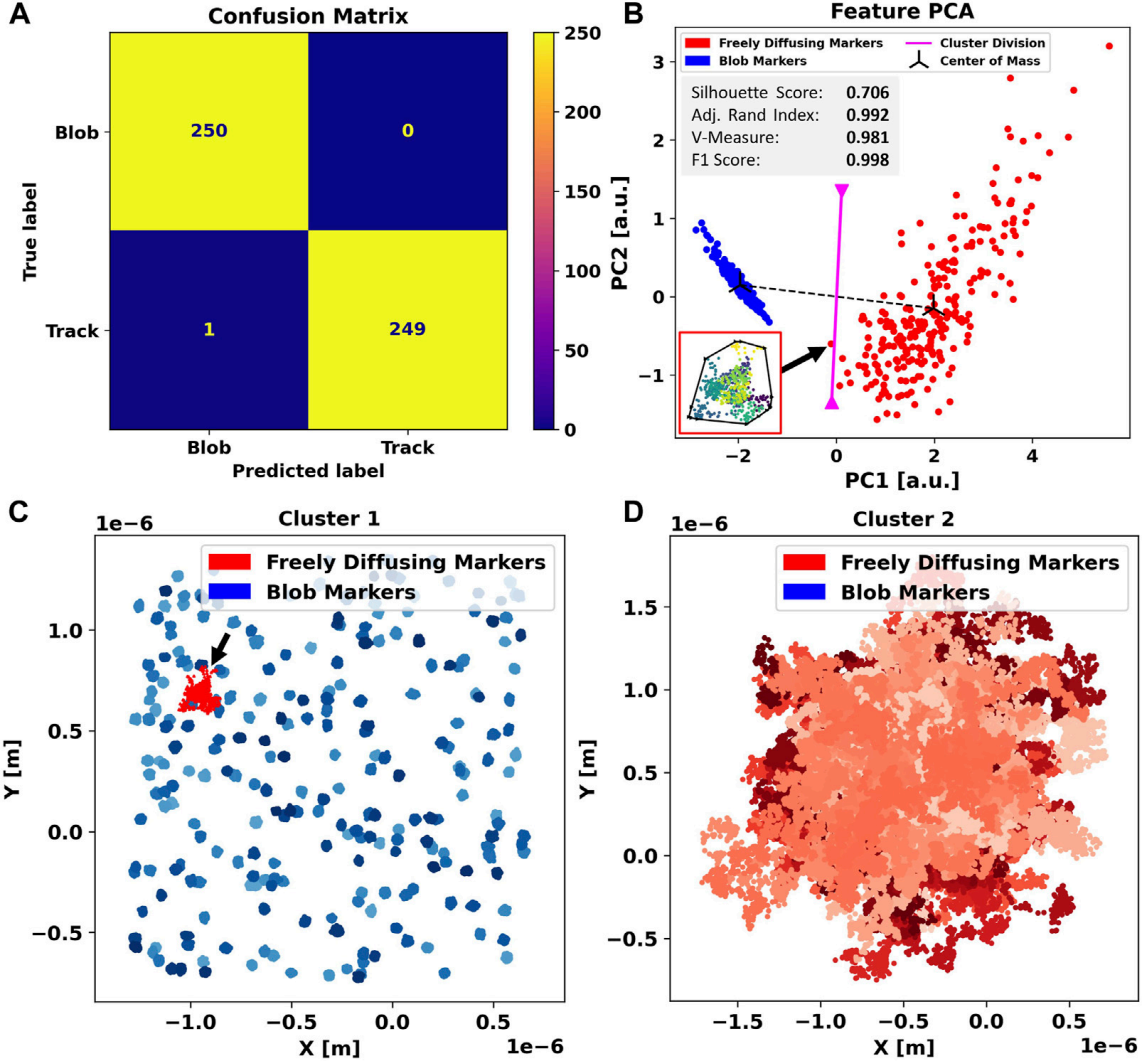
Application of Blob-B-Gone to the simulated two-dimensional dataset leads to the separation listed in the confusion matrix **(A)**. **(B)** Principal component analysis of the standardized feature space reducing the dimensionality from 5D to 2D. The gray box includes metrics (silhouette score [-1,1], adjusted Rand index [-1,1], V-measure [0,1], and F1-score [0,1]) computed for the 2D single-shot k-means clustering. Each cluster mean is marked as a center of mass in black, and the decision boundary between the assigned clusters is visualized in magenta and calculated as the normal of the segment connecting the cluster means in the middle. The black arrow connects an incorrectly labeled trace to a respective time-scale colored (dark-> bright) scatterplot. The black outline marks their convex hull. For comparison, all traces in each cluster are shown in **(C,D)** in various shades of the color corresponding to their predicted label. The color-scales are used to aid discerning individual tracks. On the left side, another black arrow highlights the incorrectly flagged blobs.

The results obtained when applied to the two-dimensional simulation dataset are shown in Figure 2. Out of 500 traces (250 free and 250 blobs), 499 are assigned the correct label and only one diffusing track was inaccurately flagged as a blob, leading to an F1-score of 0.998 (2D). The incorrectly labeled track, highlighted in red (Figure 2C), shows that this trajectory has a high concentric density of localizations and a more regular, almost circular, convex outline. Therefore, this object closely resembles a blob trajectory in terms of center sphericality, localization density, and ellipticity.

For clearer insights, we performed principal component analysis (Jolliffe and Cadima, 2016) (additional PCA plots are found in Supplementary Figure S3) reducing the feature space to two dimensions (Figure 2B), to better visualize the agglomeration of descriptors into clusters. The blob cluster (blue) appears as densely packed compared to the free (red) particles, indicating high similarity among its content. This was expected as we deliberately chose the features to highlight these geometric correlations.

We note that the cluster of free particles (red), though still visibly grouped, is spread far more than the blob cluster, indicating that the set of features chosen produced a higher variance of descriptors. This is caused by a higher variety in the distribution used to produce data, which is randomized free diffusion. Nonetheless, both populations appear clearly separated, implying that the features chosen still describe the data efficiently.

The track incorrectly flagged as an artifact is located on the far outer rim of the blob cluster, close to the diffusing one, as marked by the decision boundary in magenta (Figure 2B). We conclude that this instance of mislabeling is a direct consequence of using single-shot *k-means* and could be avoided by adjusting the boundary or switching to a *k-nearest-neighbor* algorithm. However, this would require training prior to application. The advantage of using k-means, however, lies in the fact that it is easily scalable to a large number of data points and is capable of performing single-shot clustering without annotated data, which is advantageous in terms of resources and general applicability.

**FIGURE 3:**
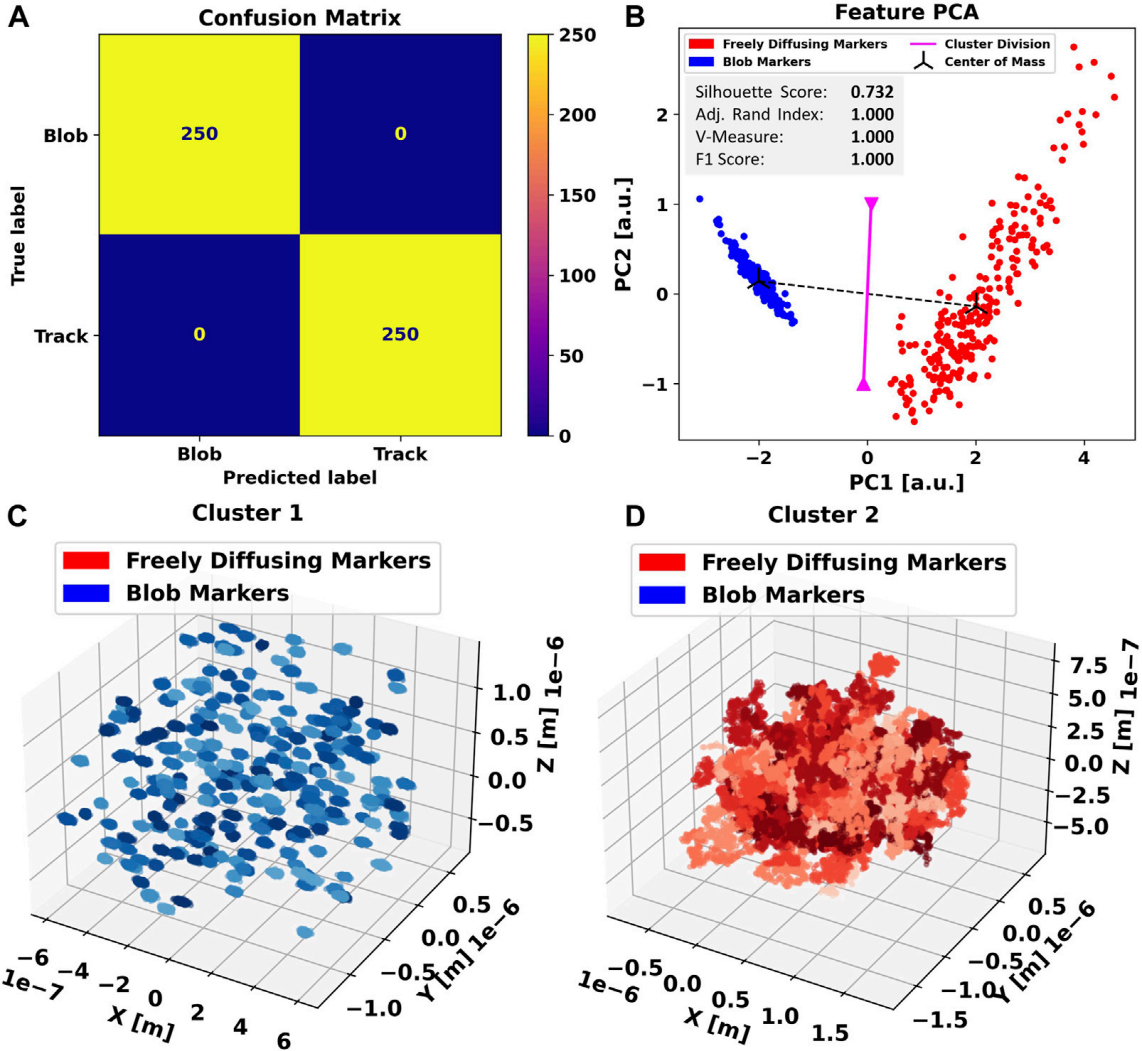
Applying Blob-B-Gone to the simulated three-dimensional dataset leads to the separation listed in the confusion matrix **(A)**. **(B)** Principal component analysis of the standardized feature space reducing the dimensionality from 5D to 2D. The gray box includes metrics (silhouette score [-1,1], adjusted Rand index [-1,1], V-measure [0,1], and F1-score [0,1]) computed for the 3D single-shot k-means clustering. Each cluster mean is marked as a center of mass in black, and the decision boundary between the assigned clusters is visualized in magenta and calculated as the normal of the segment connecting the cluster means in the middle. For comparison, all traces in each cluster are shown in **(C,D)** in various shades of the color corresponding to their predicted label. The color-scales are used to aid discerning individual tracks.

We apply the same procedure to the three-dimensional dataset. In this case, the clustering algorithm achieves a perfect separation between the blobs and freely diffusing particles, producing an exceptional F1-score of 1.0 and a diagonalized confusion matrix (Figure 3A), indicating that all data points have been assigned the correct label. In principal component analysis (Figure 3B), we further notice an even tighter clustering and clearer separation compared to the previous case (Figure 3B), indicating that the additional non-trivial dimension causes the feature ensemble to be even more representative of the particle trajectories.

Finally, we again apply Blob-B-Gone to both datasets ignoring the ground truth to evaluate the automatic annotation using the B -score introduced previously. The metric delivers a clear 37:1 ratio between blobs and free particles when applied to the clusters produced when presenting the 2D data, allowing us to unambiguously distinguish and annotate the results. Equivalent to the even sharper separation in the feature space encountered in the 3D case, we receive a 44:1 (blob: free) B -score ratio.

### 3.2 MINFLUX SPT data

After successfully applying the proposed method to simulated data in two and three dimensions, we investigate its performance on *in vitro* MINFLUX SPT data.

On average, we found approximately 20% blob artifacts per set, which renders our base dataset imbalanced. To ensure symmetric feature generation and comparability between the performances of both classes, we first equalize the population counts in the dataset by randomly picking traces from each set. However, given that during a real scenario, Blob-B-Gone will be applied to an imbalanced dataset, we also assess this case.

**FIGURE 4:**
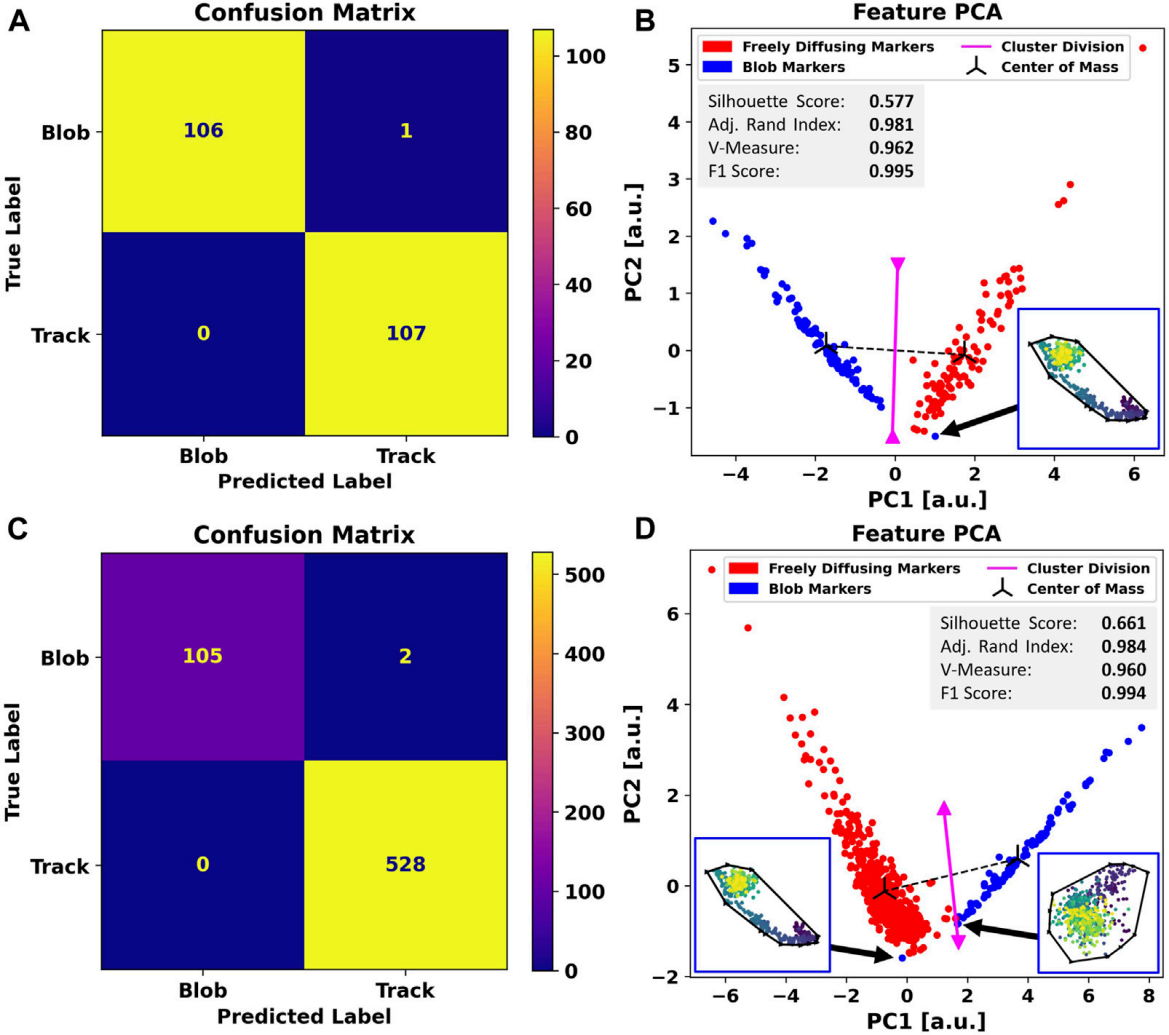
Comparison between the performance of Blob-B-Gone applied to a balanced dataset **(A,B)** as opposed to an imbalanced dataset **(C,D)**. To construct the balanced set, traces were randomly drawn from the original imbalanced hand-labeled MINFLUX SPT. The separation achieved by the algorithm is demonstrated by the confusion matrices in **(A,C)** and underlined by the principal component analysis of the standardized feature space reducing the dimensionality from 5D to 2D in **(B,D)**. The gray box includes metrics (silhouette score [-1,1], adjusted Rand index [-1,1], V-measure [0,1], and F1-score [0,1]) computed for the 2D single-shot k-means clustering. Each cluster mean is marked as a center of mass in black, and the decision boundary between the assigned clusters is visualized in magenta and calculated as the normal of the segment connecting the cluster means in the middle. The black arrows highlight the incorrectly labeled traces to respective time-scale colored (dark->bright) scatterplots. The black outline marks their convex hull.

When facing MINFLUX data with the same procedure as presented for the simulated data, the automatic annotation again yields a clear 1:33 (blob: free) B -score ratio for either set. In both cases, the same artifact has been mislabeled a free trace, leading to almost diagonalized confusion matrices (Figures 4A, C) and F1-scores of 0.995 (balanced) and 0.994 (imbalanced). Comparatively lower silhouette scores of 0.577 (balanced) and 0.661 (imbalanced) imply a narrower cluster separation, which we highlight in the respective PCAs (Figures 4B, D).

Even though the clusters are more spread out due to a higher variability in geometric structure in the experimental dataset, we still observe a clear separation between the two for the balanced set. In the case of the imbalanced dataset, both clusters reach closer to the separation border.

In total, we notice two mislabeled trajectories. One only appears in the imbalanced dataset and is likely caused by the cluster proximity. The other is a trajectory common to the balanced and imbalanced cases. We mark these outliers with black arrows and show their outline in the PCA plots of Figure 4.

The full-set-exclusive data point has a typical blob appearance, though the localizations do not appear as dense in the center, which probably causes it to be located on the outer rim of the blob cluster. It would, thus, be reasonable to assume that it originates from the dominant influence of the free trajectories.

The outlier found commonly in both sets stretches a rather long distance and convex area due to the “hooked” tail shape. The data point exhibits a significant shift along the first principal component compared to other blobs in both plots. The significant contrast, present in both balancing scenarios, could imply an inaccurately assigned ground truth label. However, the scatterplot strongly suggests that this is an artifact.

**FIGURE 5:**
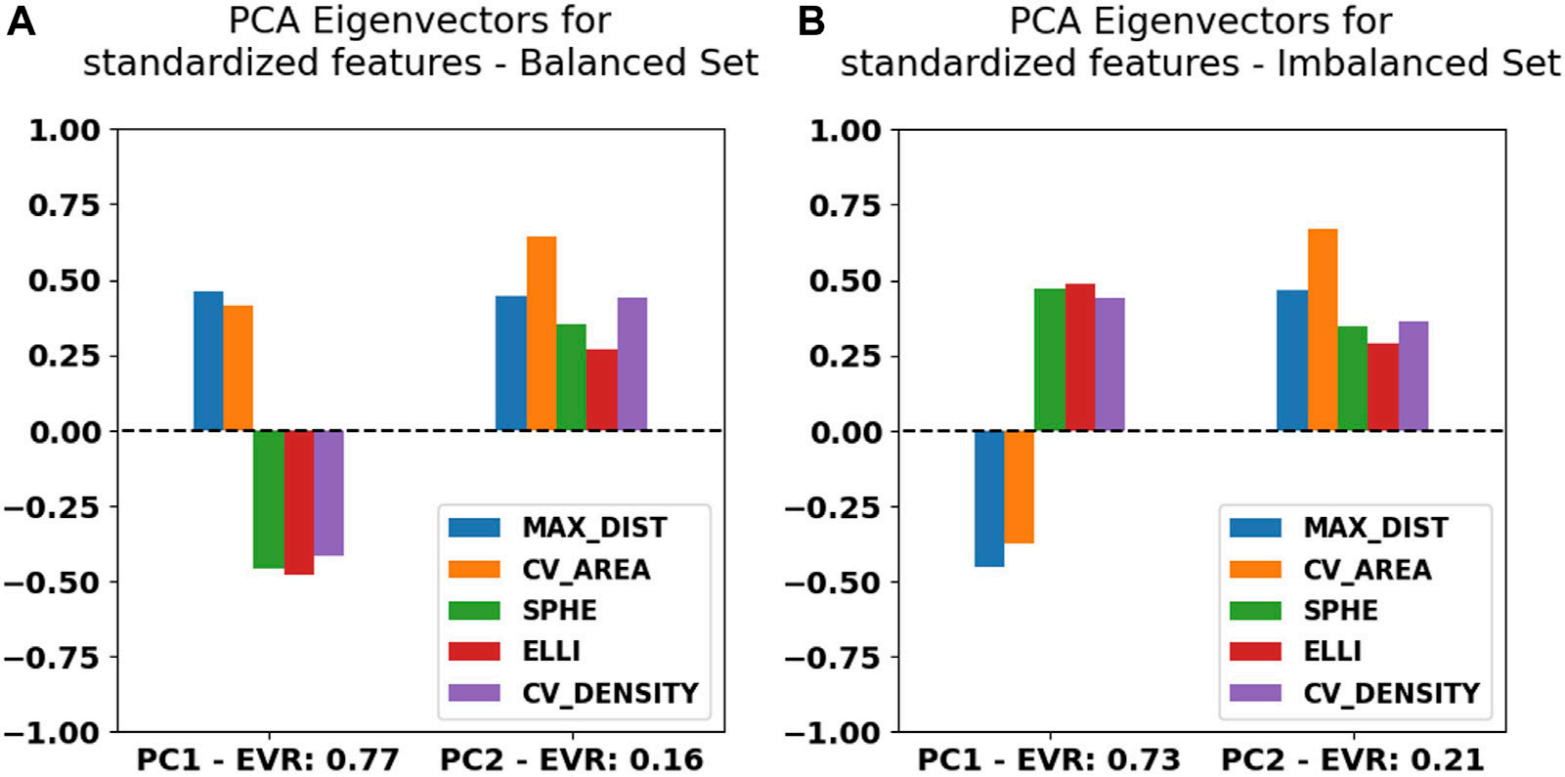
Principal component analysis eigenvector values for the first two components listed together with their explained variability ratio (EVR) for Blob-B-Gone applied to the balanced **(A)** and imbalanced **(B)** datasets of MINFLUX SPT.

The shift described previously originates from an asymmetric contribution of the *maximum distance* and *convex hull area* feature values when compared to the *sphericality*, *ellipticity*, and *convex hull density* along the first principal component, which is expected considering what the features were designed to represent and highlight. We can observe this directly within the first PCA eigenvectors (Figure 5) for either case.

**FIGURE 6:**
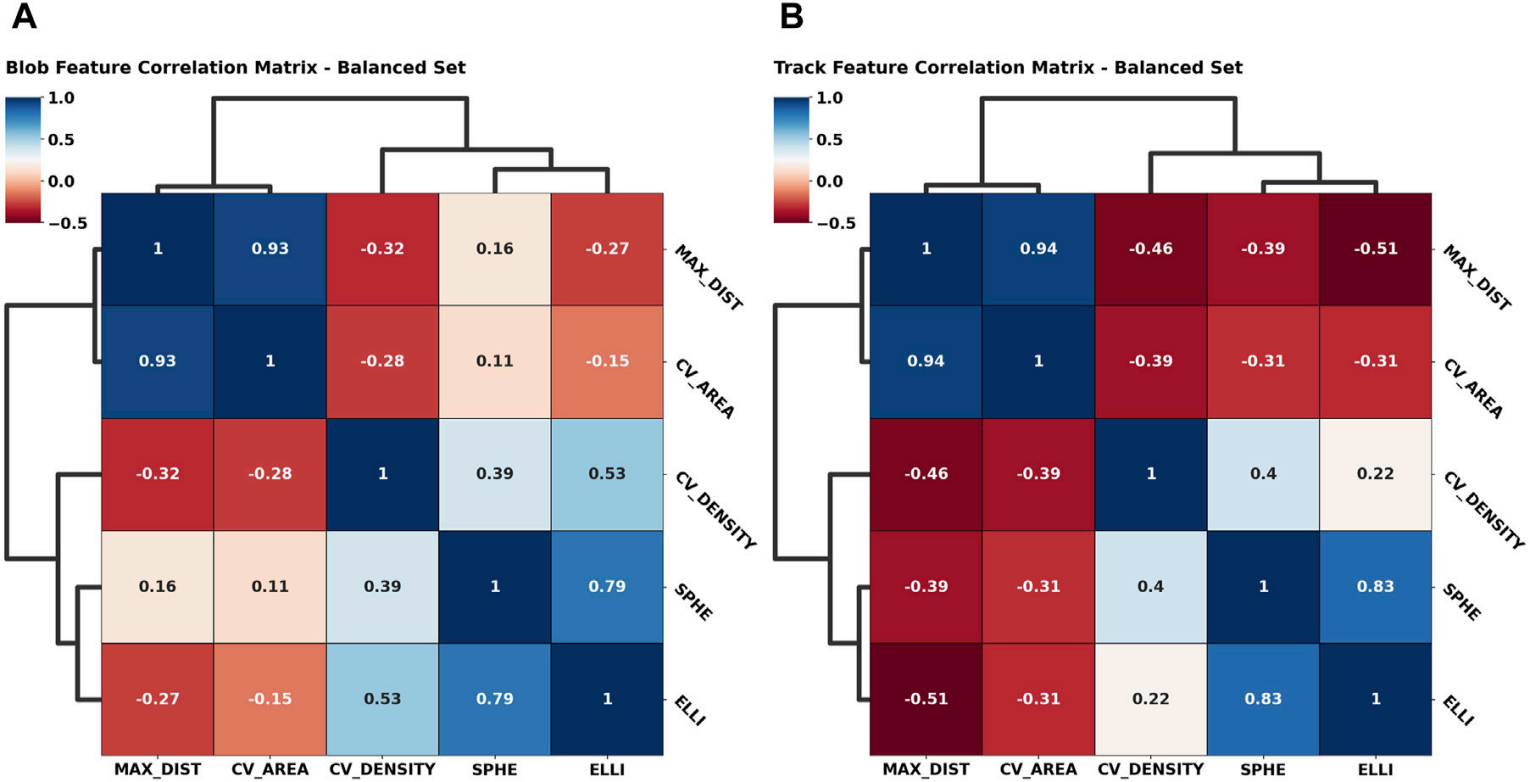
Symmetric correlation matrices of the geometric feature space color-coded by their correlation coefficient [anti-correlation (−1), correlation (1)]. A dendrogram highlights correlation clusters, i.e., over-arching generally similar behavior among feature axes. The blob feature space **(A)** is compared to the freely moving particle feature space **(B)** to underline the descriptive performance of the features presented.

Apart from the asymmetry of the first two components within the primary eigenvectors, the principal component (PC) weights for both cases investigated are remarkably similar, which is reassuring, since it underlines a certain degree of rigidity during feature space construction. In terms of absolute contribution per feature, we notice a balanced distribution among the primary PC, while it seems that the *convex hull area* contributes more significantly to the second PC. Approximately 75% of information is contained within the first principal component, as shown by the explainable variability ratio (EVR) values in Figure 5. We thus expect to find close similarity to the main correlations between the feature axes. We thus constructed cross-correlation matrices to highlight dependencies between feature axes using the PCC implementation of the *pandas* Python module (McKinney, 2010; The pandas development, 2023). In addition, we introduce correlation clustering and show the results as a cluster map using Python’s *seaborn* implementation (Waskom, 2021) (Figure 6). To ensure comparability between feature axes, we restrict ourselves to the balanced case.

The added dendrogram highlights clustered feature behavior and significance accordingly. Along the tree hierarchy, we find direct correspondence between the grouped correlation and the PCA weight distributions (Figure 5). Here, we find again the asymmetric behavior given the high correlation between the *maximum distance* and *convex hull area* feature, which in turn are anti-correlated with the *convex hull density*, *sphericality*, and *ellipticity*, as highlighted by the uppermost clade. As the observations match the intent underlying the initial feature design, we can reasonably conclude that we successfully highlighted the geometric systematic behavior. Additional correlation heatmaps for simulation and the imbalanced set can be found in Supplementary Figures S4, S5.

## 4 Discussion

In this work, we have presented Blob-B-Gone, a simple computational tool to isolate and distinguish between trajectories of freely diffusing particles and immobile (or highly constrained) particles, as detected in MINFLUX microscopy. Other artifacts, e.g., particle/stage drift, known *a priori*, should instead be addressed using similarly specialized methods prior to Blob-B-Gone to ensure accurate results. Despite the development taking place on the MINFLUX particle tracking dataset, the method can be applied to any single-particle tracking capable microscopy method. The algorithm itself merely requires two spatial coordinates per sample event, e.g., X and Y, to operate, which can originate from any particle tracking procedure.

The advantage of the approach presented herein lies in its sole reliance on the geometric properties of the point cloud of the single-particle localizations rather than other descriptors of more complex origin, such as diffusion coefficients. Moreover, we demonstrated how efficient clustering of single-particle trajectory, in certain scenarios, may be achieved without machine learning and large datasets of annotated data for training in a more straightforward and accessible way. The framework was originally developed to fully automate the classification of immobile tracers (i.e., blobs) and trajectories of diffusing markers in high-throughput single-particle tracking measurements as a big data problem. To this end, we have assessed its performance for idealized two- and three-dimensional simulated traces as well as hand-annotated *in vitro* MINFLUX SPT data on a sample routinely prepared in our laboratory.

Though this method was designed to extract blobs from rather homogeneous systems, we speculate that this approach could feasibly produce similar results in more complex scenarios, provided that more clusters are anticipated in the calculations to account for different diffusing behaviors. This is provided that a system expresses more complex movements in significant numbers to construct a balanced dataset.

Nonetheless, our method, as demonstrated by the application to the aforementioned datasets, has proven to be highly effective, with F1-scores close to 1.0 for simulation and MINFLUX data (Table 4). For simulated data, the designated descriptors enable a clean separation in the feature space (Figure 2B, 3B), with silhouette scores of 0.706 (2D) and 0.732 (3D). Though the cluster split is not as vast in the MINFLUX SPT sets (Figures 4B, D), causing smaller silhouette scores of 0.577 (balanced) and 0.661 (imbalanced), we retrieved adjusted Rand indices and V-measures close to 1.0 across all sets (Table 4). This indicates significant reliability in highlighting similarities and differences between all trace populations with a homogeneous label spread.

**TABLE 4:** Evaluation metrics across all datasets considered in this work.

Dataset	F1-score	Silhouette score	Adjusted Rand index	V-measure	B -score ratio
2D simulated	0.998	0.706	0.992	0.981	37:1
3D simulated	1.000	0.732	1.000	1.000	44:1
Balanced	0.995	0.577	0.981	0.962	33:1
Imbalanced	0.994	0.661	0.984	0.960	33:1

As an exemplary case, we investigated the primary and secondary PCA eigenvectors (Figure 5) for the balanced SPT data. These portrayed an almost uniform contribution between all features toward the final clustering, which implies that all of them successfully grasped an individual aspect of the traces presented. Additionally, we observed an expected center-asymmetry when comparing the weights of features highlighting a stretched outline to those of that describe spherically condensed point spreads. This matches the correlation pattern found between individual feature axes (Figure 6). Using feature cross-correlation matrices, we successfully identified that this behavior originates from the initial design of the clustering. This demonstrates that purely geometric descriptors of the point clouds obtained from single-particle tracking applications could provide a very practical way to classify diffusion modes.

When facing more complex structures and artifacts, it is likely that the performance of the tool as mentioned here will deteriorate due to a higher and more imbalanced variability in the trajectory datasets. This can originate from a host of different causes, such as particle–environment interaction, which will, in turn, produce a substantial overlap of clusters in the feature space we defined. Nevertheless, the performance may be restored, for example, by introducing more initial clusters, thus sensitizing the separation algorithm. *A priori* knowledge of the system, expected diffusion behavior, and, consequently, trajectory shapes, could be utilized to apply weights to features according to system-specific criteria, helping in reducing cluster overlap. Therefore, an extension of the proposed method, with labeled data, supervised training, and k-nearest-neighbor classification, could be adapted to perform a more rigid classification of any specific system. This would come at the cost of higher resources, computational time, and annotated datasets.

## Data Availability

The datasets presented in this study can be found in online repositories. The names of the repository/repositories and accession number(s) can be found at: https://github.com/Eggeling-Lab-Microscope-Software/blob-B-gone/tree/main/Example_Data.
