# Supplementary material for: Blob-B-Gone: a lightweight framework for removing blob artifacts from 2D/3D MINFLUX single-particle tracking data
Source: Front Bioinform. 2023 Nov 22;3:1268899. doi: 10.3389/fbinf.2023.1268899 (PMC10704905; doi:10.3389/fbinf.2023.1268899)
Supplement: Supplementary file 1 [file DataSheet1.PDF]

Supplementary Material

Supplementary Note

Following the described path, we simulated a set of synthetic blobs, which we display in Figure S1. For visualization purposes, we have deliberately chosen a blob with a similar misalignment of X and Y spread. In addition to that, the simulation is made drawing 239041 localizations from a two-dimensional multivariate normal distribution, the same number of positions counted in Figure 1a of the main text. A comparison reveals high similarity between real data and simulation, indicating that we found a way to generate convincing blob artifacts.

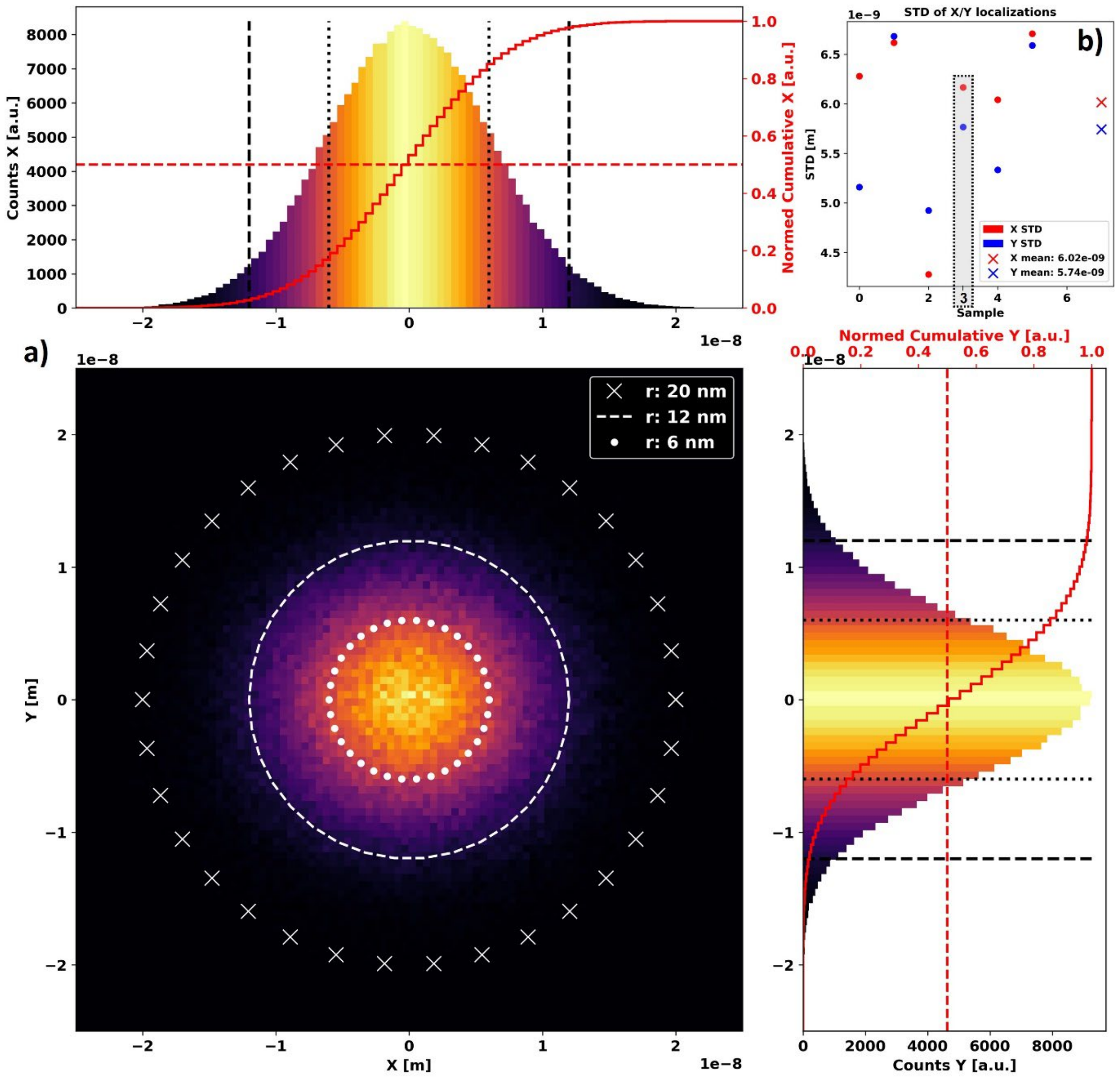

Supplementary Figure S1 - Two-dimensional histogram of 239041 simulated localizations a) drawn from a 2D multivariate normal distribution with [0,0] mean and diagonal covariance matrix composed of values extracted from MINFLUX data. Three white circles are shown additionally for reference. The X-axis profile is shown as a 1D histogram in the upper, the Y-axis profile in the right side-panel. A common full red line in both graphs marks the cumulative histogram. The dotted red line highlights 50% of all localizations. The extracted standard deviations in X and Y direction for the entire dataset is shown in b). The crosses mark the mean value of X/Y standard deviation. A gray box highlights the dataset used in the other plots.

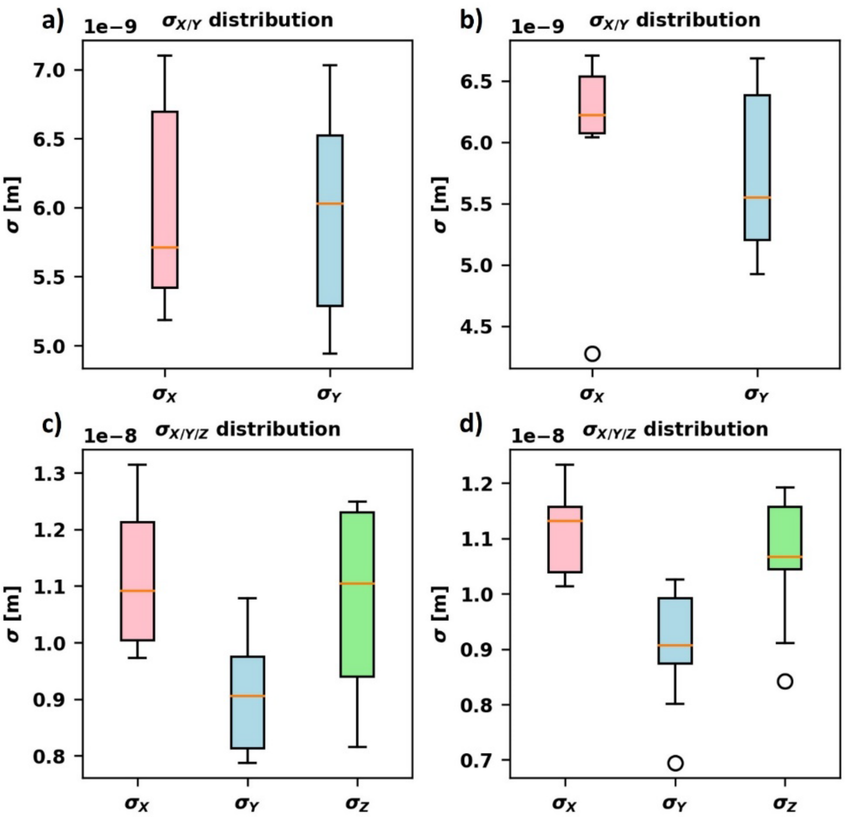

Supplementary Figure S2 – a) Standard Deviations extracted from MINFLUX 2D tracking data of GattaQuant 23nm immobile beads b) Standard deviations drawn from three-dimensional multivariate normal distributions using the extracted normal distribution of variances experienced in the data b). Panels c) and d) show the standard deviations extracted from 3D MINFLUX and simulation data equivalently.

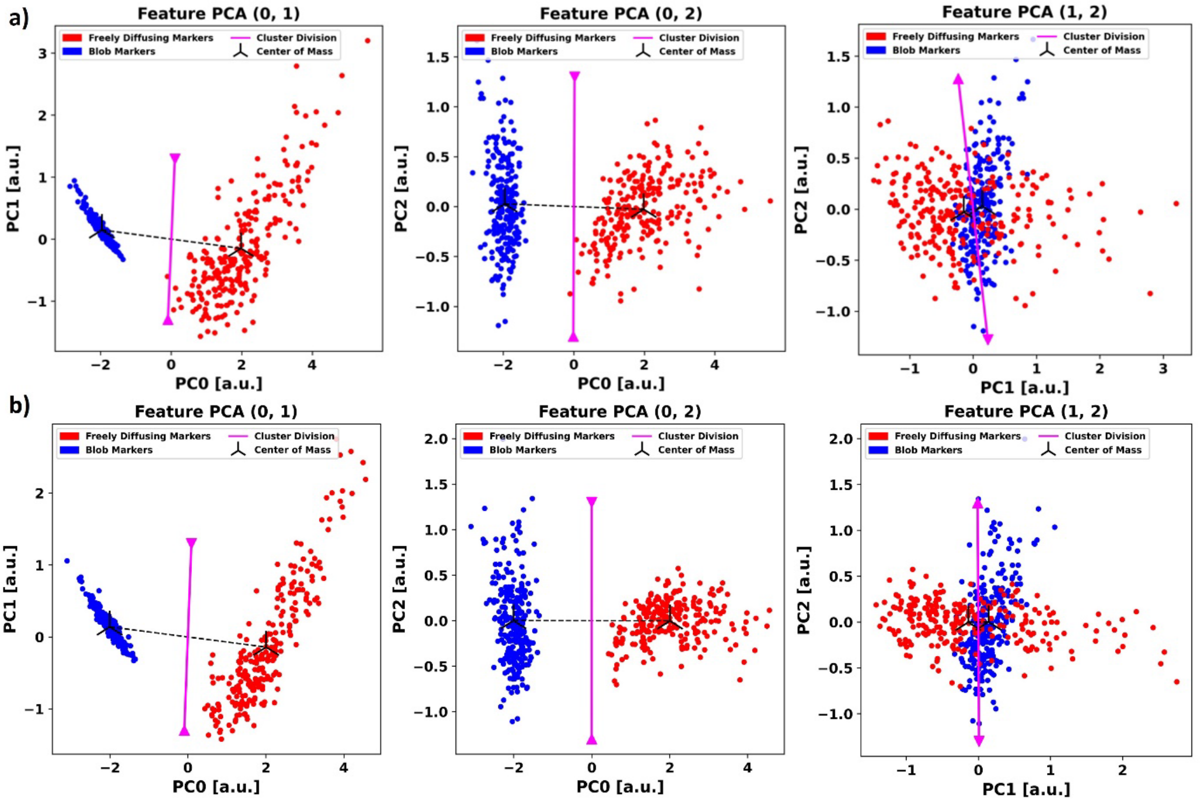

Supplementary Figure S3 – a) Principal Component Analysis of the five-dimensional feature vector used in the 2D application of Blob-B-Gone, reducing the feature-space to a three-dimensional principal component space. All points are colored according to their label assigned by single-shot *k-means++* clustering using said 5D feature space. We mark each blob center of mass and display the decision boundary in purple. Equivalently, we compute and display the respective 3D application in b).

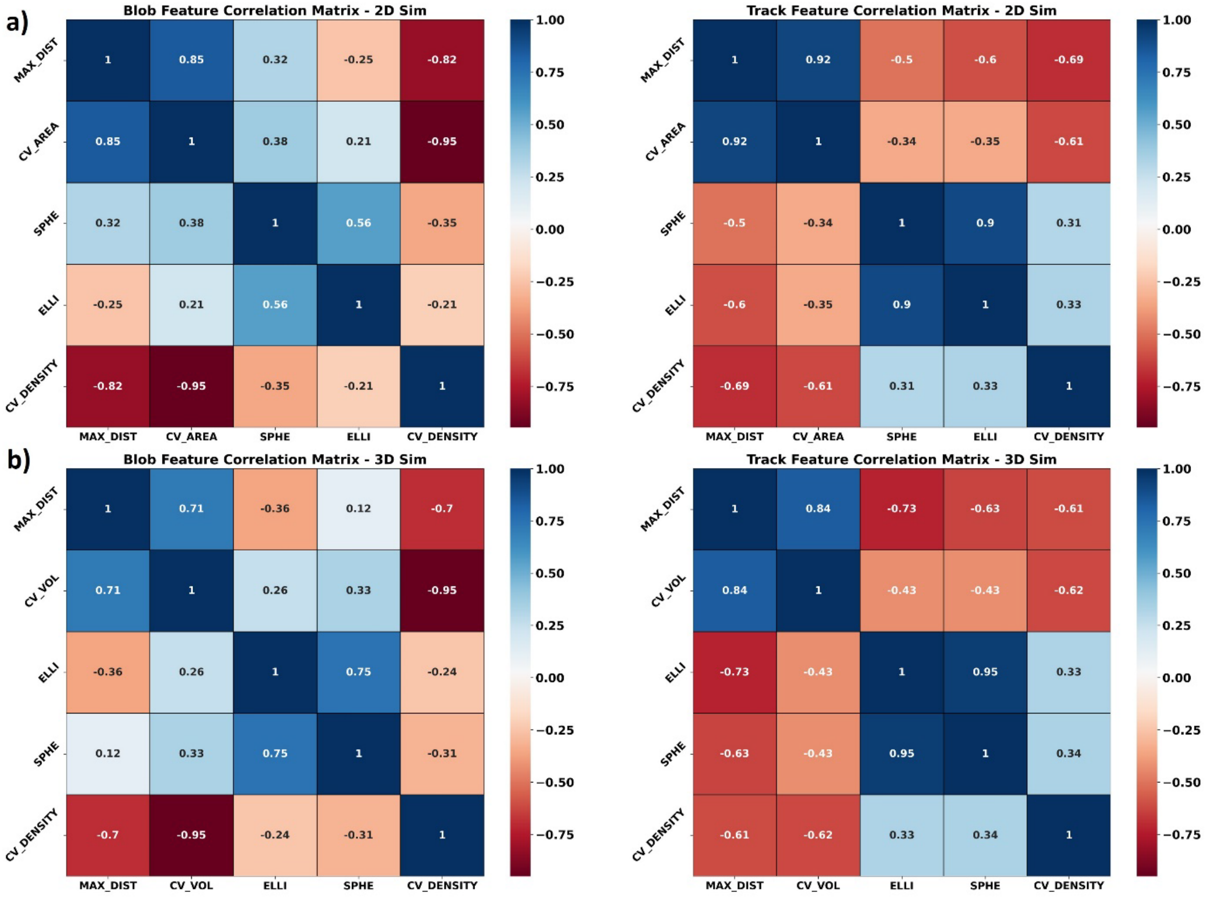

Supplementary Figure S4 *– Symmetric correlation matrices of the geometric feature space color coded by their correlation coefficient [anti-correlation (-1), correlation (1)] for 2D simulation a) and 3D simulation data b).*

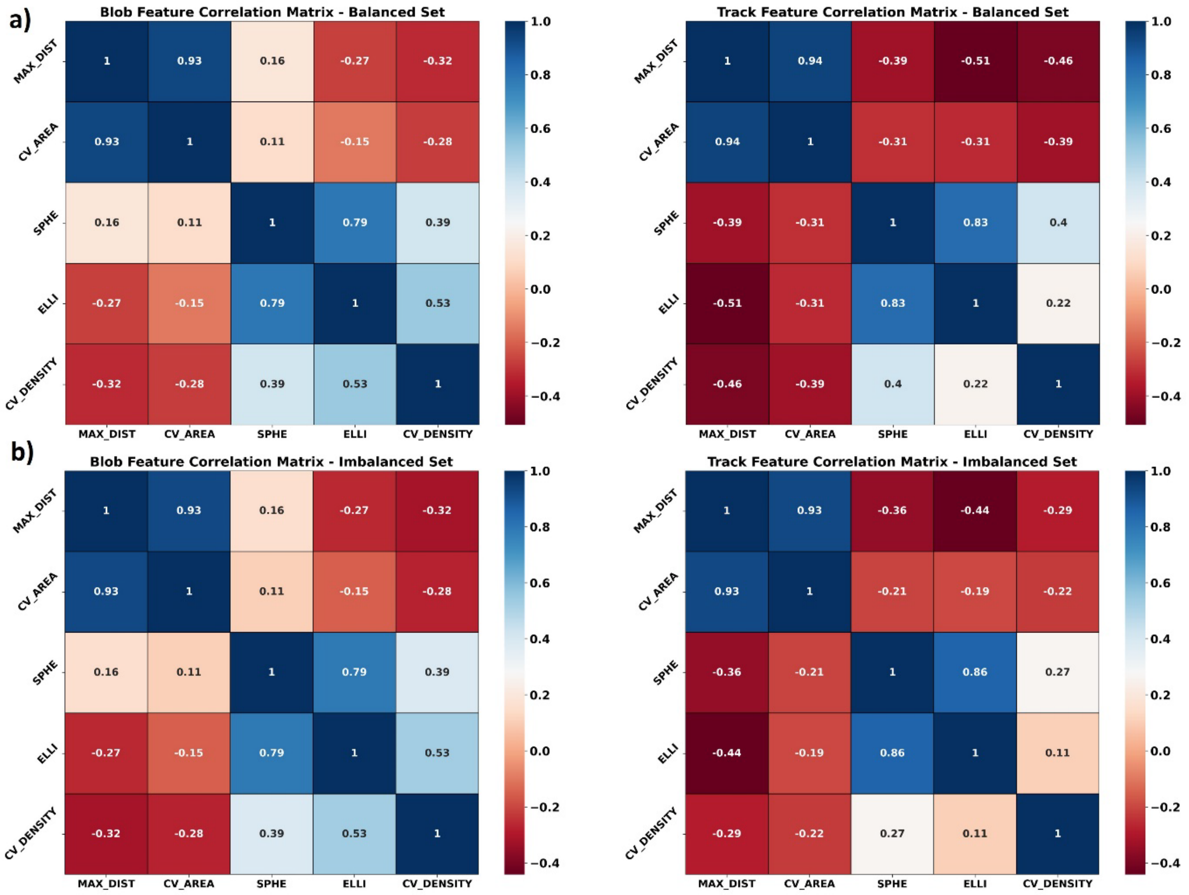

Supplementary Figure S5 *– Symmetric correlation matrices of the geometric feature space color coded by their correlation coefficient [anti-correlation (-1), correlation (1)] for the balanced a) and the imbalanced b) MINFLUX SPT data.*
